# Prevalence of Potentially Inappropriate Medications in Drug Dispensing Data of Older Adults Living in Northwest Italy

**DOI:** 10.3390/pharmacy13060184

**Published:** 2025-12-15

**Authors:** Lucrezia Greta Armando, Jacopo Luboz, Abdoulaye Diarassouba, Gianluca Miglio, Clara Cena

**Affiliations:** 1 Department of Drug Science and Technology, University of Turin, Via Pietro Giuria 9, 10125 Turin, Italy; gianluca.miglio@unito.it; 2 Struttura Complessa Farmacia Azienda USL Valle d’Aosta, Via Guido Rey 1, 11100 Aosta, Italy; iluboz@ausl.vda.it; 3 Struttura Complessa Farmacia Territoriale ASL TO4 Regione Piemonte, Via Nino Costa 43, 10034 Chivasso, Italy; adiarassouba@aslto4.piemonte.it; 4 Competence Center of Scientific Computing C3S, University of Turin, Corso Svizzera 185, 10149 Turin, Italy

**Keywords:** Beers Criteria, potentially inappropriate prescriptions, potentially inappropriate medications, drug dispensing data, older patients

## Abstract

The increasing use of multiple medications among older adults raises concerns about potentially inappropriate medications (PIMs), which are associated with adverse health outcomes and increased healthcare costs. This study aimed to assess the prevalence and types of PIMs dispensed to older adults living in Northwest Italy using real-world pharmacy claims data. An observational, retrospective analysis was conducted on anonymized drug dispensing datasets from two local health authorities, covering individuals aged 65 years or older between 2018 and 2021. PIMs were identified according to the 2019 American Geriatrics Society Beers Criteria, focusing on drugs that are inappropriate or should be used with caution in older adults or have anticholinergic properties. Over half of older adults who received medications during the study period were dispensed at least one PIM, with stable or slight increased prevalence over time with no differences by sex or region. Proton-pump inhibitors used for more than 8 weeks and paroxetine were the most common PIMs, while furosemide and sulfonylureas were also frequently reported PIMs. These findings indicate a persistently high burden of inappropriate prescribing in older adults and highlight the need for coordinated deprescribing interventions and prescriber education to promote safer, evidence-based pharmacotherapy in aging populations.

## 1. Introduction

The use of medications among older adults presents a significant challenge for healthcare systems globally due to the prevalence of polypharmacy and the risk of receiving potentially inappropriate medications (PIMs). PIMs are linked to adverse drug reactions, impaired quality of life, increased hospitalizations, and higher healthcare expenditures [1].

In Italy, demographic aging is advancing rapidly, and inappropriate prescribing has emerged as a substantial public health concern in recent years. Studies based on the use of drugs in the real-world [2,3] highlight a high prevalence of PIM use among older adults. A recent cross-sectional study from northern Italy [4] reported that almost 46.0% of older primary care patients enrolled in the study received at least one PIM, with benzodiazepines/hypnotics (19.7%) and non-steroidal anti-inflammatory drugs (NSAIDs, 6.6%) among the most frequently involved classes. In the study, PIMs were identified according to the list of potentially inappropriate prescriptions (PIPs) in older patients published by Beers and colleagues for the first time in 1991 and updated in subsequent years [5]. The Beers Criteria, as they were subsequently called, are now in their fifth version, published in 2023, and represent one of the most widely used tools to identify PIMs and to guide safer prescribing practices. They are validated, explicit prescribing indicator sets used to identify medications that merit clinical review in the context of the individual patient’s clinical history and preferences [6]. Each recommendation included in the Beers Criteria is graded according to the expected level of harm to the patient and the strength of supporting evidence. They are organized into six categories: drugs considered potentially inappropriate for individuals aged 65 years or older; drugs that may worsen certain diseases or syndromes due to drug–disease interactions in this age group; drugs that require cautious use in older adults; clinically significant drug–drug interactions that should be avoided in those ≥65 years; medications that must be avoided or dose-adjusted according to renal function in older patients; and agents with marked anticholinergic activity.

Additional regional and population-based data confirm benzodiazepines and psychotropic agents as commonly implicated in Beers-listed PIMs [7,8,9]; for instance, among Italian nursing home residents, almost all patients were prescribed at least one psychotropic PIM [8,9].

In recent years, the analysis of real-world data, particularly drug dispensing data, has become an increasingly important tool for assessing the appropriateness of prescriptions, both internationally and in Italy [10]. Analyses based on pharmacy claims allow researchers to evaluate medication use in large populations, monitor trends over time, and identify high-risk prescribing patterns. These approaches offer a reliable and scalable method for evaluating PIM prevalence at both regional and national levels; they can also provide the information needed to guide interventions aimed at promoting safer prescribing in older adults [11,12,13].

This study thus aims to assess the prevalence of PIM dispensing among older adults living in northwest Italy. By focusing on real-world pharmacy claims, we seek to enhance our understanding of local prescribing practices to propose targeted strategies to reduce PIM use and promote appropriate evidence-based prescribing among aging populations.

## 2. Materials and Methods

### 2.1. Data Source

This was an observational, retrospective study which used drug dispensing data to investigate the presence of PIMs among medications dispensed from local pharmacies. As explained in previous studies [11,12,13], Italian drug dispensing datasets, included in databases collected for administrative and accounting purposes by local health authorities (LHAs), were transmitted anonymously to the authors. LHAs selected for the analysis were the LHA Torino 4 (ASL TO4) and the LHA Valle d’Aosta (AUSL VdA); these are neighboring areas located in northwest Italy providing healthcare to part of the Piedmont region (the ASL TO4 has approximately 500,000 inhabitants) and to the entire Valle d’Aosta Region (approximately 125,000 inhabitants).

Data collected encompass the period from 1 January 2018 to 31 December 2021 and capture medications prescribed by general practitioners (GPs) to patients living in the LHAs and reimbursed by the Italian National Health Service (NHS). Each record includes anonymized patient identifiers, date of birth, sex, date of dispensation, medication name, active ingredient, Anatomical Therapeutic Chemical (ATC) classification code [14], and the number of packages dispensed. These records were further linked to the local registry of deaths to retrieve mortality data, where applicable.

This study adhered to the EU General Data Protection Regulation (2016/679), ensuring full compliance with data privacy regulations. All data were anonymized at the source and researchers did not have access to any personally identifiable information. The Ethics Committees responsible for the two LHAs were notified about the study, complying with the national guideline for the classification and conduct of observational studies on medicines [15], and data exchange was regulated by previously signed research agreements between the LHAs and the authors.

### 2.2. Study Population

The study population comprised individuals over 65 years of age or who turned 65 during the study period (1 January 2018–31 December 2021) who received dispensations of medications reimbursed by the Italian NHS. Patients with at least one dispensation of a PIM according to the Beers Criteria, version 2019 [16] represented the eligible population. The 2019 update of the Beers criteria was chosen because it covers most of the study period. PIMs based on clinical conditions or on the presence of specific conditions were excluded from the analysis as clinical data was not available. PIMs based on conditions related to the drug (i.e., dosage > 325 mg/day, duration ≥ 8 weeks) were evaluated for each patient and included in the study dataset if relevant. Individuals who died before 31 December 2021 were excluded from the analysis.

The drug dispensing datasets containing relevant PIMs of enrolled patients were analyzed separately for the two LHAs to describe the use of PIMs in the regions investigated.

Patients were stratified according to the type of PIMs dispensed, as well as by their gender and age calculated at the end of the study period. Three age groups were selected according to the classification provided by the Italian National Institute of Health [17]: young elderly (65–74 years old), elderly (75–84 years old), very elderly (≥85 years old).

### 2.3. PIMs

A table version of the 2019 update of the Beers Criteria was prepared with the Microsoft Excel program linking each drug to its ATC code. Drug dispensing datasets of eligible patients were then cross-referenced with this table using the ATC code as a common key to enable identification of PIMs.

According to the classification of the 2019 Beers Criteria [16], PIMs were classified based on their type into medications that are potentially inappropriate in most older adults (Table 2 in ref. [16]); medications to be used with caution in older adults (Table 4 in ref. [16]); medications with strong anticholinergic properties (Table 7 in ref. [16]). Among these PIMs, some were excluded because it was not possible to assess the presence of specific conditions in the patients. For other PIMs, the conditions for their applicability were assessed as follows: concomitant use with other drug classes, dispensation duration, dosage limits, pharmaceutical form and administration route. The complete list of PIMs analyzed is included in Supplementary Materials, Table S1. Selected PIMs included in Table 4 in ref. [16] were potentially inappropriate in the study population as they can cause syndrome of inappropriate anti-diuretic hormone (SIADH) or hyponatremia, while PIMs according to Table 2 in ref. [16] were potentially inappropriate due to various reasons (see Table S1 for further details). The other PIMs in the Beers Criteria were excluded as they require some clinical information in order to be considered relevant for the patients.

Due to the nature of drug dispensing data, certain simplifications have been adopted to assess the relevance of PIMs:

- For dosage limits, the unit dose of dispensed medications was considered rather than the prescribed dose (missing data).
- For concomitant uses, a period of 3 months was considered to check the co-dispensing of the drug classes concerned, in accordance with methodologies reported elsewhere [18].
- For uses > 8 weeks, the distance between consecutive dispensing events was measured, and drugs dispensed more than 56 days apart and within 180 days were considered PIMs.
- Drug use was considered chronic when there were 5 or more dispensing events of the drug in a year. This is a definition commonly used in drug utilization research [19].
- Pharmaceutical forms and administration routes were derived from medication names.

Despite being removed by the 2019 Beers Criteria, in this study, dispensations of ticlopidine were considered potentially inappropriate. Ticlopidine was included in the Beers Criteria, version 2015, as a drug to be avoided in older patients [20], then removed from the 2019 update as it was no longer available on the US market (Table 8 in ref. [16] —medications/criteria removed since 2015 American Geriatrics Society Beers Criteria) [16]. In Italy, however, ticlopidine is marketed and reimbursed by the NHS and was therefore included in the analysis. Other drugs removed from the 2019 update of the Beers Criteria were not included in this analysis as they belong to Table 3 in ref. [16] (drug-disease or drug-syndrome interactions that may exacerbate the disease or syndrome, excluded due to lack of clinical data) or are not recorded in drug dispensing data collected by LHAs.

### 2.4. Analysis

Patient demographics and PIMs dispensed were summarized using descriptive statistics. Year prevalence of PIM use was measured for each type of PIMs as the number of patients with selected PIMs compared to the number of older adults who received medication dispensing during the same period. PIM frequencies were reported annually as the number of patients with that specific PIM, combining drugs with the same mechanism of action (ATC level 4).

All statistical analyses were performed using R (version 4.0.5; https://cran.r-project.org/; accessed on 7 July 2025). The following R packages and their dependencies were employed: lubridate, dplyr and ggplot2.

## 3. Results

### 3.1. Study Samples

A total of 115,962 and 25,873 subjects aged 65 years and over received at least one dispensation of a medication reimbursed by the NHS during the study period in the ASL TO4 and the AUSL VdA, respectively. According to the population statistics for those years [21,22], these correspond to the 23.0% of the entire population of the ASL TO4 (83.0% of older people) and to the 21.0% of the population of the AUSL VdA (84.9% of older people).

The number of older adults with at least one PIM during the study period was 65,807 for the ASL TO4 (56.7% of older adults with drug dispensations) and 14,359 for the AUSL VdA (55.5% of older adults with dispensations). The general characteristics of the study samples are summarized in Table 1. No differences were found between the two regions. The proportion of patients with at least one PIM and with PIMs classified according to their type remains stable over the years. Patients were mostly females with a median age ranging from 76 to 77 years and a median number of PIMs of 7 per year.

**Figure 1:**
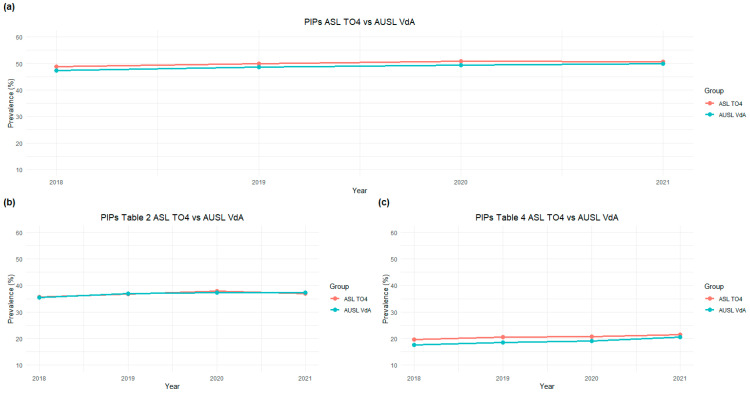
Annual prevalence of older patients with PIMs compared to the total older patients with drug dispensations. Panel (**a**) shows the total PIMs; panel (**b**) shows PIMs according to Table 2 in ref. [16] (drugs to avoid); panel (**c**) shows PIMs according to Table 4 in ref. [16] (drugs to be used with caution). Abbreviations: PIPs, Potentially Inappropriate Prescriptions.

Figure 1 shows the annual prevalences of patients with PIMs during the study period for the two regions, from which a slight increase is observed. PIMs are shown both as total PIMs and classified according to their different type. No differences in the prevalence of use of PIMs were found between the two regions, between males and females or between different age groups (Supplementary Materials, Figures S1–S6).

### 3.2. Temporal Trends According to PIM Type: Drugs to Avoid and Drugs to Be Used with Caution

The following tables summarize the most frequent annual PIMs divided according to type. As shown in Table 2, the most frequent PIM (according to Table 2 in ref. [16]—drugs to avoid) was represented by proton-pump inhibitors (PPIs) used for more than 8 weeks, followed by paroxetine or sulfonylureas, which were potentially inappropriate due to their anticholinergic effects and higher risk of hypoglycemia, respectively. Temporal trends for PPIs remained constant over the years without differences between the two regions. In both regions, the use of sulfonylureas slightly decreased, while that of other PIMs remained almost unchanged, representing a small portion of the study population (less than 3% of older patients).

Table 3 shows the most frequent PIMs to be used with caution in older patients. Furosemide ranks first and its use increased in both regions; it is considered potentially inappropriate in older patients as it may exacerbate syndrome of inappropriate antidiuretic hormone secretion or hyponatremia. A reduction in the use of aldosterone antagonists and of fixed combinations of minor diuretics and potassium-sparing agents was observed, while the use of tramadol remains constant in both the ASL TO4 and the AUSL VdA. Some differences between the two regions were observed in the lower ranks: mirtazapine, antipsychotics, and hydrochlorothiazide use were prevalent only in the AUSL VdA region and demonstrated a slight increase in prevalence over the years, while minor diuretics and fixed combinations of furosemide and potassium-sparing agents were used more frequently in the ASL TO4 region.

Ticlopidine was considered potentially inappropriate in older patients in the previous versions of the Beers Criteria as more effective and safer alternatives were available (Table 2—drugs to avoid) [20]. While it is no longer marketed in the US, in the Italian regions analyzed, a limited number of patients received dispensations of ticlopidine prescribed by the GP during the study period: 1679 patients (1.4%) living in the ASL TO4 and 240 patients (0.9%) living in the AUSL VdA, with a median number of dispensations of ticlopidine of 6 per year, ranging from 1 to 21. The use of ticlopidine remained almost unchanged over the years, without differences between the two regions (Supplementary Materials, Figure S7).

### 3.3. Use of Drugs with Strong Anticholinergic Properties by Older Adults

The use of drugs with strong anticholinergic properties according to Table 7 in ref. [16] demonstrated slight differences in the number of patients considered for the two LHAs and increased slightly over the years. Older patients who received dispensations for drugs with high anticholinergic activity during the study period accounted for 11.7% of the study sample of the ASL TO4 (7678 patients) and for 9.5% of the AUSL VdA study sample (1362 patients). In 2021, the most dispensed drugs with strong anticholinergic effects were the same for both regions: paroxetine, amitriptyline and olanzapine. Moreover, 26.5% (2033 patients) and 24.6% (335 patients) of patients with anticholinergic drugs for the ASL TO4 and the AUSL VdA, respectively, received 5 or more dispensations in 2021 and were therefore considered chronic users. The total number of chronic users of strong anticholinergics for the two LHAs was 2368, with a median age of 75.0 years (IQR 70.0–81.0) and a preponderance of female patients (1738, 73.4%). Their characteristics are summarized in Table 4.

Almost all chronic users of drugs with strong anticholinergic properties received at least one dispensation of an antidepressant listed in Table 7 in ref. [16], while 12.5% received dispensations for antipsychotics with strong anticholinergic properties. Other drugs with anticholinergic effects were dispensed to less than 10.0% of the study sample. The most used chronic anticholinergics were paroxetine (1691 patients, 71.4%), olanzapine (248, 10.5%) amitriptyline (211, 8.9%), oxybutynin (149, 6.3%) and clomipramine (137, 5.8%). All these drugs have an anticholinergic cognitive (ACB) scale according to the Aging Brain Program of the Indiana University Centre [23] of 3, which expose patients to a greater risk of delirium and, in the presence of multiple anticholinergics, increase the risk of death in older patients. Within this study sample, only 12 patients (0.5%) received 3 different drugs with strong anticholinergic effects during 2021, with an ACB scale of 9, while 146 (6.2%) had 2 different anticholinergics for a total ACB scale of 6.

## 4. Discussion

In this study, the presence of PIMs was evaluated in drug dispensing data of adults aged 65 or older living in two distinct regions in northwest Italy. It was highlighted that more than half of older patients with medications dispensed during the 4-year study period (2018–2021) received at least one PIM, and that the prevalence of PIM remained stable or slightly increased over the years. No differences were observed between the two regions examined and between males and females.

The use of the Beers Criteria as a tool to evaluate the burden of inappropriate prescribing confirmed the persistence of therapy-related issues in the older population under analysis. Nevertheless, it is well known that application of explicit criteria cannot account for the complexity of patient sub-populations, nor should it be taken as definitive indication for the choice of excluding a specific drug from an individual patient therapy. The results obtained from this type of analysis should anyway generate focused alerts to prescribers and help guide their clinical decision.

Therefore, this study highlights two main issues, including the widespread use of PPIs and paroxetine among the elderly; these issues should be addressed and prioritized to optimize medication prescribing in Northwest Italy. PPIs and paroxetine are considered potentially inappropriate in older patients according to Table 2 in ref. [16], 2019 update; moreover, paroxetine has been recognized as an agent with strong anticholinergic properties and is also included in Table 7 in ref. [16].

Our findings can be compared with previous Italian and European studies investigating PIM prevalence through dispensing or prescribing data. For example, Galimberti et al. [2] used a validated, national list of PIMs in elderly (the ERD list) [24], including recommendations extracted from the 2015 Beers Criteria, to investigate the use of medications by older adults living in the Piedmont Region. The authors reported a decreasing trend in PIM prevalence from 2012 to 2018, with 30.7% of older adults still exposed to at least one PIM in 2018. Although the trend in PIM prevalence is opposite to that found in our study, the percentage of patients with at least one PIM in 2018 is comparable, accounting for 34.0% of older patients living in the area of the ASL TO4 within the Piedmont Region. The differences observed may be due to the different areas investigated (entire region vs. single LHA), the different years analyzed (2012–2018 vs. 2018–2021), and the various PIM lists used (2015 ERD list vs. 2019 Beers Criteria). Similar to our observations, Galimberti and colleagues found no major sex differences, and PPIs were consistently among the most frequently dispensed PIMs, together with diclofenac. Other studies conducted in Europe [25,26] confirmed the predominance of PPIs, NSAIDs, and benzodiazepines as common PIMs in the elderly. The extensive use of PPIs in older adults deserves particular attention. These drugs are among the most commonly prescribed by both GPs and specialists, with prescription rates reaching 55.0–63.0% upon discharge in various Italian hospitals [27,28]. Despite their effectiveness for specific indications, long-term and inappropriate use of PPIs has been associated with more or less serious adverse outcomes, including increased risk of rehospitalization, all-cause mortality, functional decline, *Clostridium difficile* infections, fractures, cardiovascular events, drug–drug interactions via CYP450 metabolism and impaired absorption of calcium, vitamin B12, iron and magnesium [27,28]. Elli et al. [27] found that nearly two-thirds of older adults discharged from internal medicine and geriatric wards in Italy (62.9%) received a PPI, with over 60.0% of these prescriptions classified as inappropriate according to the recommendations defined by the Italian Medicine Agency (AIFA), which partly overlap with the Beers Criteria [29,30]. Importantly, 26.0% of such patients were re-hospitalized within one year. Similar findings have been reported by Schepisi et al. [28] and by Casula et al. [31], who confirmed the widespread and inappropriate use of PPIs in older adults regardless of educational interventions targeting prescribers. Despite the availability of international deprescribing guidelines since the early 2000s and their extensive dissemination in Italy in recent years [32], we did not observe a declining trend in inappropriate PPI use, which is increasing in both regional analyses, suggesting that deprescribing practices remain limited in routine healthcare, while they should be encouraged with ad hoc interventions.

Paroxetine also emerged as a commonly dispensed PIM in both the ASL TO4 and the AUSL VdA, with almost constant prevalence of use in the years 2018–2021. This is noteworthy given its well-documented anticholinergic properties, which are considerably stronger compared to other selective serotonin reuptake inhibitors (SSRIs) [33,34]. Previous studies [34,35] have linked paroxetine exposure to increased sedation, constipation, weight gain, cognitive decline and risk of dementia and SIADH, as well as to the classical anticholinergic side effects, such as constipation, urinary retention, increased intraocular pressure, blurred vision, dry mouth, dry eyes, flushing and hyperthermia. Although it is unclear whether paroxetine increases the risk of dementia in older adults compared to other SSRIs [33], its pharmacological profile suggests increased vulnerability to anticholinergic adverse effects, and caution should be used when prescribed in older patients, particularly those with cognitive impairment. It also causes numerous drug interactions due to action on the CYP450 superfamily [36]. Indeed, paroxetine is a strong inhibitor of P450 3A4 isoenzyme, which metabolizes approximately half of prescribed drugs [37]; moreover, it represents the strongest inhibitor of P450 2D6 isoenzyme among all antidepressants, responsible for the metabolism of medications such as antipsychotics, tricyclic antidepressants, class IC antiarrhythmics, β-adrenergic agents, trazodone and dextromethorphan [34]. Despite this, paroxetine has been the second most prescribed antidepressant after sertraline in the general population from 2018 to 2023, according to the annual reports on drug use by the AIFA [38].

Two other PIMs that deserve attention are sulfonylureas and furosemide. Sulfonylureas are potentially inappropriate according to Table 2 in ref. [16] because they can cause severe prolonged hypoglycemia and SIADH in older adults compared to other hypoglycemic agents. In spite of the well-documented risks associated with the use of sulfonylureas, their use remained remarkably high within the ASL TO4, both in treatment-naïve patients and as second-line therapy after metformin, as demonstrated by a previous study conducted using the ASL TO4 drug dispensing data [13]. In response, targeted educational initiatives have been promoted among GPs within the LHA to encourage the transition toward safer and more evidence-based glucose-lowering agents.

Finally, diuretics, particularly furosemide, were present in more than half of the population analyzed in both regions. While diuretics are listed in the 2019 Beers Criteria as PIMs in older adults because of their association with hyponatremia and the risk of SIADH, this finding underscores that such criteria should always be interpreted within a clinical context rather than applied rigidly. In many cases, such as in patients with heart failure or volume overload, the use of furosemide may be clinically appropriate and even necessary, despite its potential adverse effects. These observations highlight the importance of individualized assessment and clinical judgment when applying prescribing quality indicators to complex older populations.

### 4.1. Study Strengths and Limitations

A major strength of this study lies in the use of large and comprehensive datasets including all reimbursed drug dispensations in an entire Italian region and one LHA in Piedmont. This extensive coverage allowed for the identification of PIMs in older adults at the population level and provided valuable evidence on the magnitude and patterns of PIM exposure among older adults in northwest Italy, providing a foundation for planning future interventions to improve prescription appropriateness.

The main limitation of the study is the lack of clinical information and patient outcomes, such as diagnoses, laboratory results, indications for treatment, and reporting of adverse effects. Consequently, some medications classified as PIMs may have been appropriately prescribed based on individual clinical conditions or may not have led to negative effects. Therefore, our findings should be interpreted as identifying potentially prevalent cases of inappropriate prescribing, providing a useful basis for further investigations rather than definitive assessments of clinical appropriateness. This is a common limitation of retrospective, observational studies carried out with administrative electronic health records collected for accounting purposes; however, such studies enrich the literature on possible medication-related risks for older adults and provide further insight of inappropriate prescribing in different regions.

### 4.2. Future Directions

To optimize deprescribing to reduce PIMs and to help healthcare professionals identify and manage medication-related risks, LHA stakeholders could consider a medication review process that involves the use of digital tools that integrate the Beers Criteria together with other validated recommendations, such as alternative treatments to PIMs [39]. The implementation of these tools, the Beers Criteria, and evidenced based information into a patient care pathway has the potential to reduce medication errors and improve the quality of life [40]. The LHAs should further consider the implementation of targeted prescriber educational programs aimed at improving polypharmacy. Future research will be warranted to determine if these strategies and programs will be effective measures to reduce PIMs in older patients in Northwestern Italy and other regions.

## 5. Conclusions

Despite study limitations, this retrospective study based on anonymized administrative drug dispensing data demonstrated that a persistent prevalence of inappropriate prescribing among older adults remains in Northwest Italy. Future studies should be considered to determine if coordinated deprescribing interventions and prescriber education will promote safer evidence-based pharmacotherapy in this patient population.

## Figures and Tables

**Table 1:** General characteristics of the study samples.

	Total	2018	2019	2020	2021
**Area 1: ASL TO4**					
Total, n (%)	65,807	44,397 (63.4)	48,491 (69.2)	50,731 (72.4)	54,028 (77.1)
Males, n (%)	26,552 (40.3)	17,234 (38.8)	19,181 (39.6)	20,380 (40.2)	21,742 (40.2)
Age, median [IQR]	77 [72–82]	78 [73–83]	77 [72–82]	76 [71–82]	76 [71–82]
Age range, n (%)					
65–74 years	28,391 (40.5)				
75–84 years	27,076 (38.6)				
≥85 years	10,340 (14.8)				
PIMs per patient, median [IQR]	19 [6–37]	8 [4–13]	7 [3–12]	7 [4–11]	7 [4–11]
Patients with PIMs to avoid, n (%) ^a^	46,928 (71.3)	32,363 (72.9)	35,721 (73.7)	37,741 (74.4)	39,535 (73.2)
Patients with PIMs to be used with caution, n (%) ^b^	34,618 (52.6)	17,916 (40.4)	19,848 (40.9)	20,768 (40.9)	22,841 (42.3)
Patients with anticholinergic PIMs, n (%) ^c^	7678 (11.7)	3975 (51.5)	4197 (54.7)	4304 (56.1)	4685 (61.0)
**Area 2: AUSL VdA**					
Total, n	14,359	9662 (62.9)	10,198 (69.0)	11,251 (73.3)	12,036 (78.4)
Males, n (%)	5796 (40.4)	3770 (39.0)	4190 (39.5)	4510 (40.1)	4879 (40.5)
Age, median [IQR]	77 [72–83]	78 [73–83]	77 [72–83]	77 [72–82]	77 [71–82]
Age range (years)					
65–74 years	6055 (39.4)				
75–84 years	5928 (38.6)				
≥85 years	2376 (15.5)				
PIMs per patient, median [IQR]	20 [6–39]	7 [3–13]	7 [3–12]	7 [4–12]	7 [4–12]
Patients with PIMs to avoid, n (%) ^a^	10,519 (73.3)	7241 (74.9)	8058 (76.0)	8542 (75.9)	9000 (74.8)
Patients with PIMs to be used with caution, n (%) ^b^	7318 (51.0)	3589 (37.1)	4056 (38.3)	4366 (38.8)	4948 (41.1)
Patients with anticholinergic PIMs, n (%) ^c^	1362 (9.5)	585 (42.9)	691 (50.7)	745 (54.7)	863 (63.4)

^a^ PIMs according to Table 2 in ref. [16]. ^b^ PIMs according to Table 4 in ref. [16]. ^c^ PIMs according to Table 7 in ref. [16].

**Table 2:** PIMs dispensed to >2% of the study population (first six ranks) according to Table 2 in ref. [16] (drugs to avoid). “Patients (%)” refers to the percentage of patients with dispensations for the corresponding drug/drug class compared to the total number of patients with PIMs belonging to Table 2 in ref. [16] for each year.

	2018		2019		2020		2021	
	ASL TO4	AUSL VdA	ASL TO4	AUSL VdA	ASL TO4	AUSL VdA	ASL TO4	AUSL VdA
Rank 1	PPI	PPI	PPI	PPI	PPI	PPI	PPI	PPI
Patients (%)	**86.5**	**86.6**	**87.9**	**88.1**	**88.6**	**88.9**	**88.3**	**88.0**
Rank 2	Paro	SU	Paro	SU	Paro	Paro	Paro	Paro
Patients (%)	**8.3**	**6.4**	**7.9**	**5.6**	**7.6**	**5.3**	**7.9**	**5.6**
Rank 3	SU	Paro	SU	Paro	NSAID	SU	Amio	SU
Patients (%)	**3.6**	**5.1**	**2.8**	**5.2**	**2.5**	**5.1**	**2.3**	**4.9**
Rank 4	NSAID	Amio	NSAID	Amio	SU	Amio	TCA	Amio
Patients (%)	**2.7**	**2.7**	**2.6**	**2.7**	**2.4**	**2.9**	**2.3**	**3.4**
Rank 5	TCA	NSAID	TCA	TCA	TCA	TCA	NSAID	TCA
Patients (%)	**2.7**	**2.5**	**2.5**	**2.4**	**2.2**	**2.3**	**2.3**	**2.8**
Rank 6	-	TCA	-	NSAID	-	-	-	-
Patients (%)	-	**2.5**	-	**2.3**	-	-	-	-

Abbreviations: PPI, proton pump inhibitors; Paro, paroxetine; SU, sulfonylureas; NSAID, nonsteroidal anti-inflammatory drugs; Amio, amiodarone; TCA, tricyclic antidepressants.

**Table 3:** PIMs (first six ranks) according to Table 4 in ref. [16] (drugs to be used with caution). “Patients (%)” refers to the percentage of patients with dispensations for the corresponding drug/drug class compared to the total number of patients with PIMs belonging to Table 4 in ref. [16] for each year.

	2018		2019		2020		2021	
	TO4	VdA	TO4	VdA	TO4	VdA	TO4	VdA
Rank 1	Furo	Furo	Furo	Furo	Furo	Furo	Furo	Furo
Patients (%)	**53.4**	**50.4**	**57.7**	**53.6**	**59.1**	**55.1**	**60.8**	**56.1**
Rank 2	M diu ass	Aldo	M diu ass	Aldo	Aldo	Aldo	Aldo	Aldo
Patients (%)	**17.0**	**15.0**	**13.8**	**16.4**	**14.0**	**17.1**	**14.8**	**20.1**
Rank 3	Aldo	Tra	Aldo	Tra	M diu ass	Tra	M diu ass	Tra
Patients (%)	**11.7**	**14.0**	**13.0**	**12.6**	**10.8**	**11.9**	**9.9**	**11.2**
Rank 4	Tra	M diu ass	M diu	M diu ass	Tra	Mirta	Tra	Mirta
Patients (%)	**9.5**	**12.3**	**9.5**	**8.7**	**7.5**	**8.5**	**7.1**	**8.6**
Rank 5	M diu	Mirta	Tra	Mirta	M diu	M diu ass	Furo ass	Antip
Patients (%)	**6.6**	**7.7**	**8.7**	**8.1**	**7.0**	**6.3**	**7.0**	**7.4**
Rank 6	Furo ass	Hydro	Furo ass	Hydro	Furo ass	Antip	M diu	M diu ass
Patients (%)	**5.8**	**7.0**	**6.5**	**7.8**	**6.7**	**5.9**	**6.5**	**5.9**

Abbreviations: Furo, furosemide, torsemide; M diu ass, association of minor diuretics and potassium-sparing agents; Aldo, aldosterone antagonists; Tra, tramadol; M diu, minor diuretics; Mirta, mirtazapine; Furo ass, association of furosemide and potassium-sparing agents; Antip, antipsychotics; Hydro, hydrochlorothiazide.

**Table 4:** Characteristics of older patients with chronic dispensations for drugs with strong anticholinergic effects (year 2021).

Total, n (%)	2368
Males, n (%)	630 (26.6)
Age, median [IQR]	75.0 [70.0–81.0]
Age range, n (%)	
65–74 years	1088 (45.9)
75–84 years	985 (41.6)
≥85 years	295 (12.5)
Dispensations of chronic anticholinergics per patient, median [IQR]	6 [6–8]
Frequency of chronic anticholinergics, n (%)	
Antidepressants	1975 (83.4)
Antipsychotics	297 (12.5)
Drugs for urinary frequency and incontinence	176 (7.4)
Trihexyphenidyl	5 (<1)
Disopyramide	2 (<1)
Hydroxyzine	1 (<1)

## Data Availability

The data presented in this study are available on request from the corresponding author due to agreements on the use of data between the authors and the Local Health Authorities that provided the data (research agreements).

## Supplementary Materials

The following supporting information can be downloaded at: https://www.mdpi.com/article/10.3390/pharmacy13060184/s1, Table S1: PIMs dispensed to the study population according to the 2019 Beers Criteria (PIMs with assessable conditions or without conditions). Figure S1: Prevalence of patients with dispensations of PIMs (total) according to region of residence and gender; Figure S2: Prevalence of patients with dispensations of PIMs (total) according to region of residence and age group; Figure S3: Prevalence of patients with dispensations of PIMs to avoid in elderly (Table 2 in ref. [16]) according to region of residence and gender; Figure S4: Prevalence of patients with dispensations of PIMs to avoid in elderly (Table 2 in ref. [16]) according to region of residence and age group; Figure S5: Prevalence of patients with dispensations of PIMs to be used with caution in elderly (Table 4 in ref. [16]) according to region of residence and gender; Figure S6: Prevalence of patients with dispensations of PIMs to be used with caution in elderly (Table 4 in ref. [16]) according to region of residence and age group; Figure S7: Prevalence of use of ticlopidine according to region of residence.
