# Supplementary material for: Prevalence of Potentially Inappropriate Medications in Drug Dispensing Data of Older Adults Living in Northwest Italy
Source: Pharmacy (Basel). 2025 Dec 15;13(6):184. doi: 10.3390/pharmacy13060184 (PMC12736479; doi:10.3390/pharmacy13060184)
Supplement: Supplementary file 1 [file pharmacy-13-00184-s001.zip › pharmacy-3960768-supplementary.pdf]

Supplementary Materials

Table S1. PIMs dispensed to the study population according to the 2019 Beers Criteria (PIMs with assessable conditions or without conditions).

**Beers criteria Table 2 – drugs to avoid in elderly**			**Beers criteria Table 4 – drugs to be used with caution in elderly**			**Beers criteria Table 7 – strong anticholinergics**		
**ATC code**	**Active ingredient**	**Rationale[a]**	**ATC code**	**Active ingredient**	**Rationale[a]**	**ATC code**	**Active ingredient**	**Rationale[a]**
A02BC01	Omeprazole	(Use for >8 weeks) Risk of *C. difficile* infection, bone loss, and fractures, unless for high-risk patients.	C03AA03	Hydrochlorothiazide	May exacerbate or cause syndrome of inappropriate anti-diuretic hormone secretion (SIADH) or hyponatremia.	C01BA03	Disopyramide	Highly anticholinergic.
A02BC02	Pantoprazole		C03BA04	Chlortalidone		G04BD04	Oxybutynin	
A02BC03	Lansoprazole		C03BA08	Metolazone		G04BD07	Tolterodine	
A02BC04	Rabeprazole		C03BA11	Indapamide		G04BD08	Solifenacin	
A02BC05	Esomeprazole		C03CA01	Furosemide		N04AA01	Procyclidine	
A03BB01	Butylscopolamine	Highly anticholinergic, uncertain effectiveness.	C03CA04	Torsemide		N05AA01	Chlorpromazine	
A03BB05	Cimetropium bromide		C03DA01	Spironolactone		N05AB06	Trifluoperazine	
A03FA01	Metoclopramide	Risk of extrapyramidal effects, unless for gastroparesis for >12 weeks.	C03DA02	Potassium canrenoate		N05AH02	Clozapine	
A10AB01	Insulin (human)	(Insulin regimens containing only rapid-acting insulin) Higher risk of hypoglycaemia.	C03DA03	Canrenone		N05AH03	Olanzapine	
A10AB04	Insulin lispro		C03DA04	Eplerenone		N05BB01	Hydroxyzine	
A10AB05	Insulin aspart		C03EA01	Hydrochlorothiazide and potassium-sparing agents		N06AA04	Clomipramine	
A10AB06	Insulin glulisine		C03EA14	Butizide and potassium-sparing agents		N06AA06	Trimipramine	

A10BB01	Glibenclamide	Higher risk of severe prolonged hypoglycaemia.	C03EB01	Furosemide and potassium-sparing agents		N06AA09	Amitriptyline	
A10BB12	Glimepiride		N02AX02	Tramadol		N06AA10	Nortriptyline	
C01BA03	Disopyramide	May induce heart failure, strong anticholinergic.	N03AF01	Carbamazepine		N06AB05	Paroxetine	
C01BD01	Amiodarone	Greater toxicity than other antiarrhythmics used in atrial fibrillation.	N03AF02	Oxcarbazepine		R06AD02	Promethazine	
C08CA05	Nifedipine	(Immediate release) Risk of hypotension, myocardial ischemia.	N05AA01	Chlorpromazine				
G03BA03	Testosterone	Risk of cardiac problems, unless for symptomatic hypogonadism.	N05AA02	Levomepromazine				
G03CA03	Oestradiol	(Systemic use) Carcinogenic potential, lack of cardio- and cognitive protection effect.	N05AB02	Fluphenazine				
G03CX01	Tibolone		N05AB06	Trifluoperazine				
G03FA01	Norethisterone and oestrogen		N05AC01	Periciazine				
G03FA11	Levonorgestrel and oestrogen		N05AD01	Haloperidol				
G03FA14	Dydrogesterone and estrogen		N05AD06	Bromperidol				
G03FA17	Drospirenone and oestrogen		N05AE05	Lurasidone				
G03FB05	Norethisterone and oestrogen		N05AF05	Zuclopenthixol				
G03FB08	Dydrogesterone and oestrogen		N05AG02	Pimozide				
G03FB09	Levonorgestrel and oestrogen		N05AH02	Clozapine				

G03FB12	Nomegestrol and oestrogen		N05AH03	Olanzapine	
H01AC01	Somatropin	Risk of oedema, arthralgia, carpal tunnel syndrome, gynecomastia, impaired fasting glucose, and small impact on body composition, unless for established growth hormone deficiency.	N05AH04	Quetiapine	
L02AB01	Megestrol	Higher risk of thrombotic events and death, minimal effect on weight.	N05AH05	Asenapine	
M01AB15	Ketorolac	Higher risk of gastrointestinal bleeding, peptic ulcer disease, and acute kidney injury.	N05AH06	Clotiapine	
M01AC01	Piroxicam	(Oral, chronic use, except for patients taking gastroprotective agents) Higher risk of gastrointestinal bleeding or peptic ulcer, may increase blood pressure and induce kidney injury.	N05AL03	Tiapride	
M01AC06	Meloxicam		N05AL05	Amisulpride	
M01AE01	Ibuprofen		N05AL07	Levosulpride	
M01AE03	Ketoprofen		N05AN01	Lithium	
M01AX01	Nabumetone		N05AX08	Risperidone	
N03AA02	Phenobarbital	High rate of physical dependence, tolerance to sleep benefits, higher risk of overdose.	N05AX12	Aripriprazole	
N04AA01	Trihexyphenidyl	More effective agents available.	N05AX13	Paliperidone	

N03AE01	Clonazepam	Higher risk of sensitivity, decreased metabolism, higher risk of cognitive impairment, delirium, falls, fractures, and motor vehicle crashes.
N05BA06	Lorazepam	
N05BA12	Alprazolam	
N05CD05	Triazolam	
N05BB01	Hydroxyzine	Highly anticholinergic.
N05CF02	Zolpidem	Risk of delirium, falls, fractures, increased hospitalizations, motor vehicle crashes, minimal improvement in sleep latency and duration.
N06AA04	Clomipramine	Higher risk of cerebrovascular accident, cognitive decline, and mortality, unless in schizophrenia or bipolar disorder.
N06AA09	Amitriptyline	
N06AA10	Nortriptyline	
N06AB05	Paroxetine	
**Removed from the 2019 update**		
B01AC05	Ticlopidine	Safer, effective alternatives available.

[a]Adapted from the 2019 Beers Criteria.

N05AX15	Cariprazine
N05AX16	Brexpiprazole
N06AX11	Mirtazapine

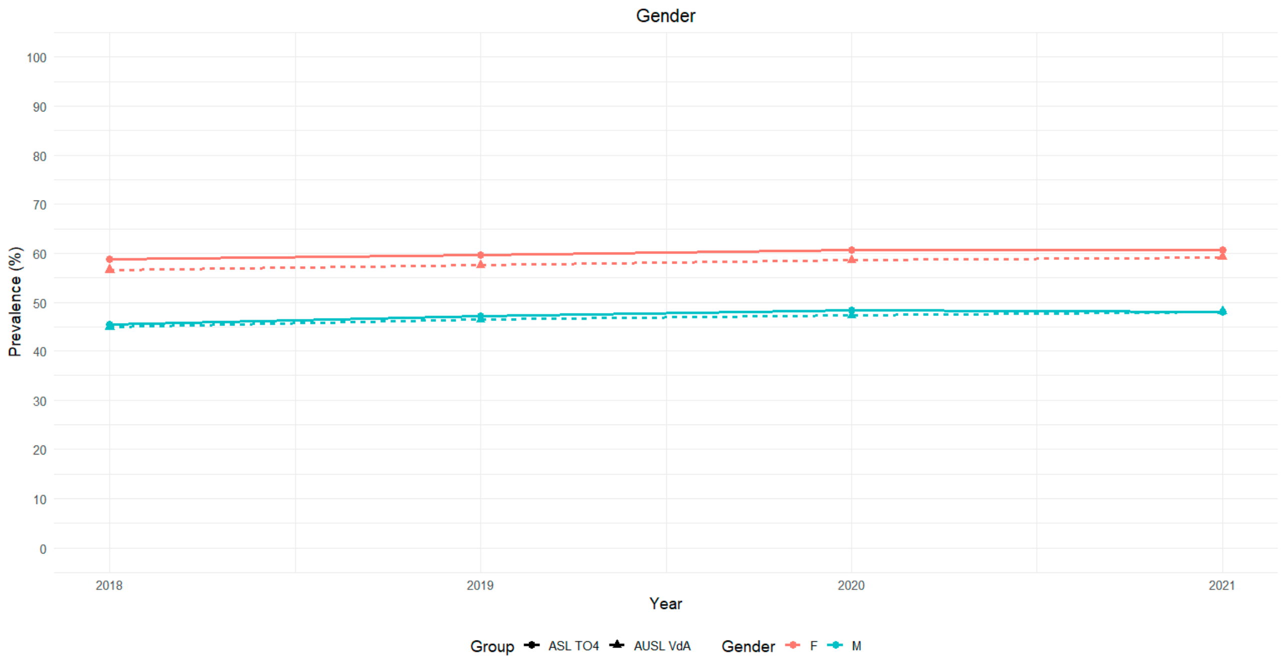

Figure S1. Prevalence of patients with dispensations of PIMs (total) according to region of residence and gender.

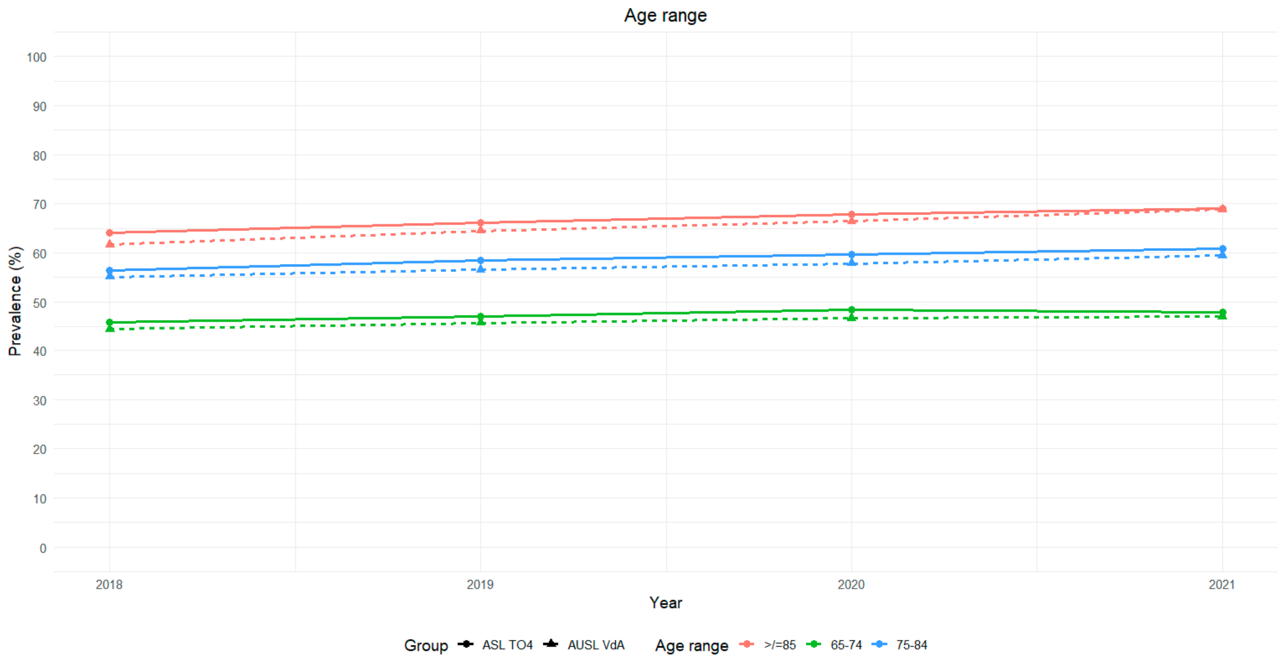

Figure S2. Prevalence of patients with dispensations of PIMs (total) according to region of residence and age group.

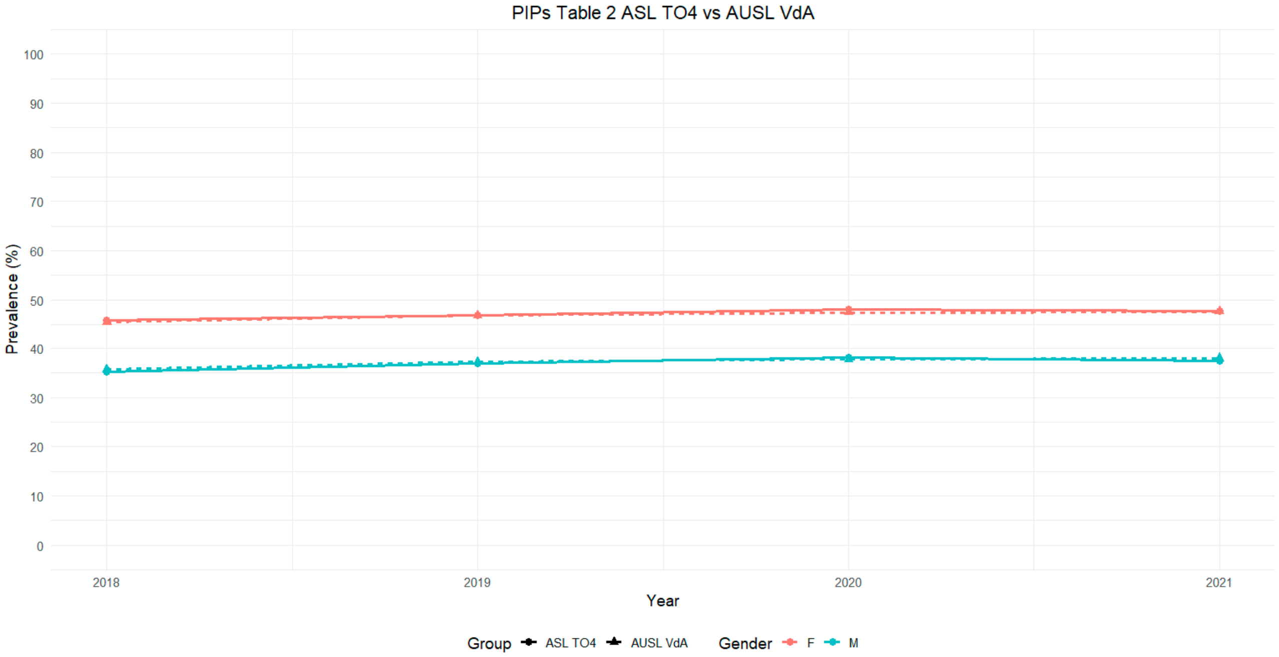

Figure S3. Prevalence of patients with dispensations of PIMs to avoid in elderly (Table 2 in ref. [16]) according to region of residence and gender.

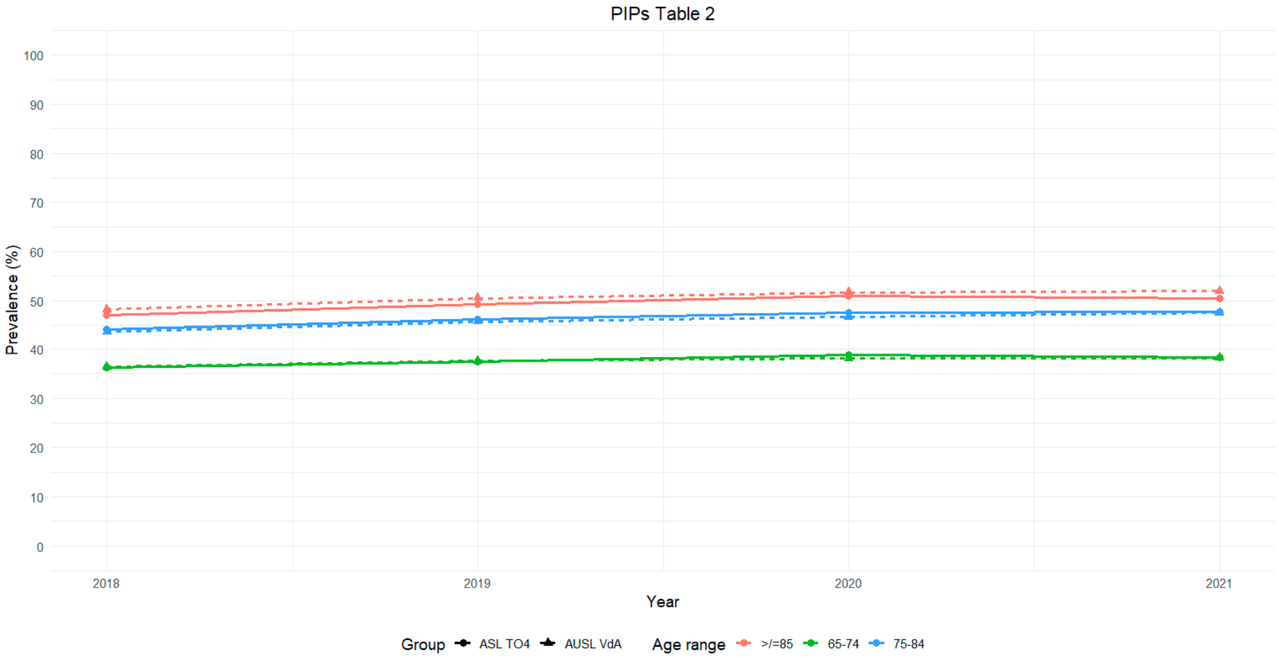

Figure S4. Prevalence of patients with dispensations of PIMs to avoid in elderly (Table 2 in ref. [16]) according to region of residence and age group.

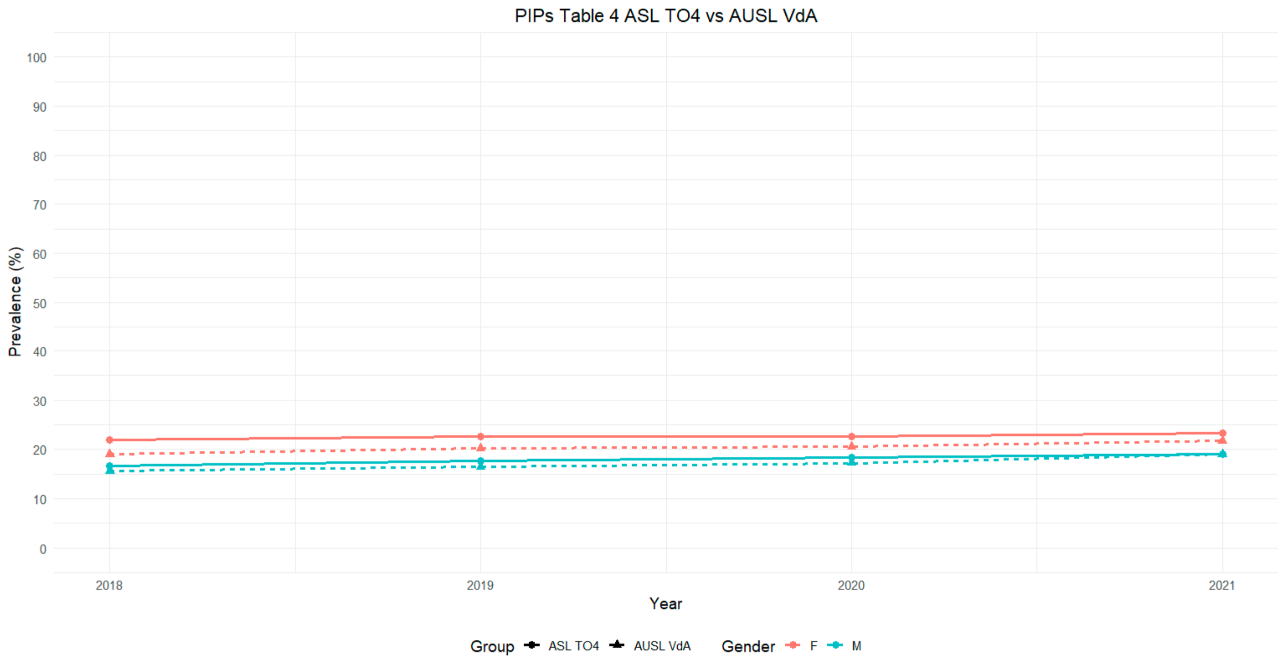

Figure S5. Prevalence of patients with dispensations of PIMs to be used with caution in elderly (Table 4 in ref. [16]) according to region of residence and gender.

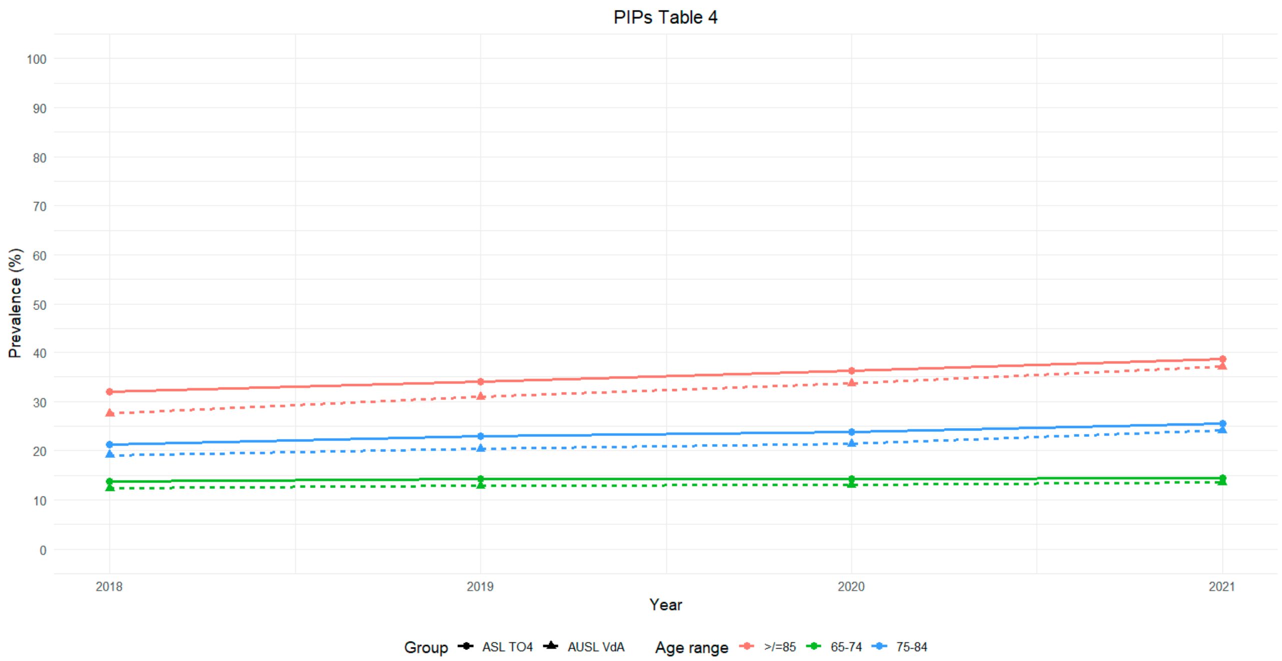

Figure S6. Prevalence of patients with dispensations of PIMs to be used with caution in elderly (Table 4 in ref. [16]) according to region of residence and age group.

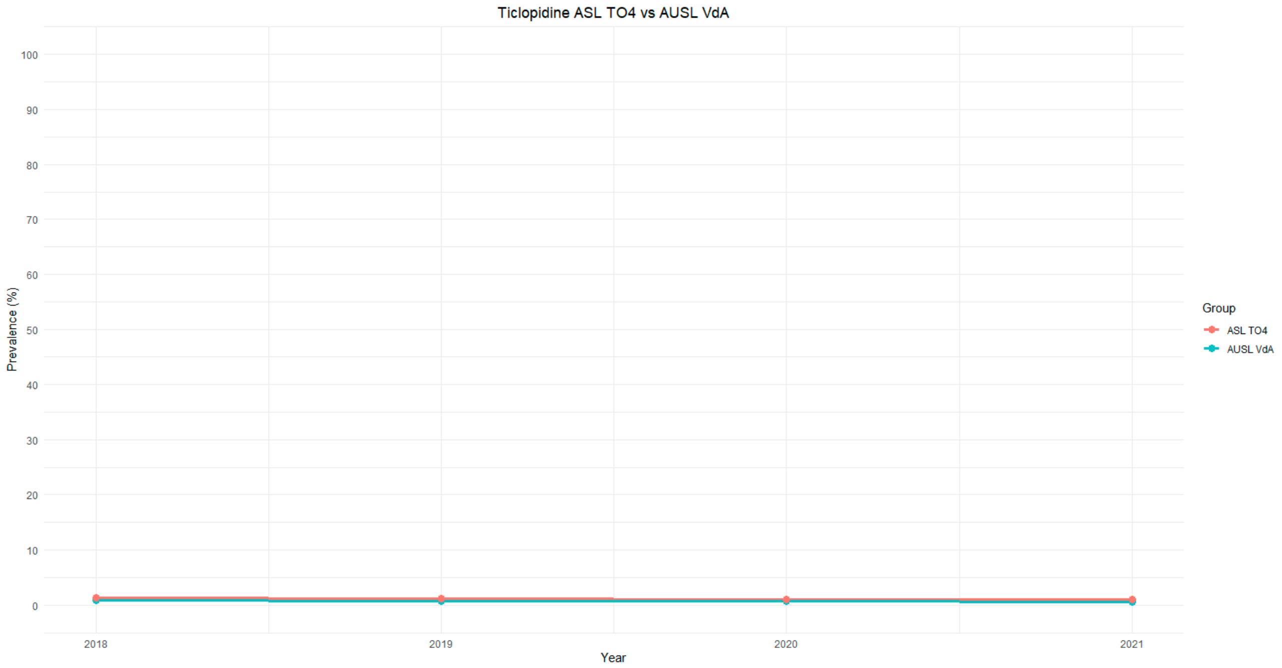

Figure S7. Prevalence of use of ticlopidine according to region of residence.
